# Anticoagulation is the answer in treating noncritical COVID-19 patients

**DOI:** 10.1515/med-2021-0354

**Published:** 2021-10-05

**Authors:** Azad A. Kabir

**Affiliations:** Department of Research and Innovation, Doctor Ai LLC, 1120 Beach Blvd, Biloxi, MS 39530, United States of America; Department of Internal Medicine, Jackson Hospital, 1725 Pine St, Montgomery, AL 36106, United States of America

**Keywords:** anticoagulation, noncritical COVID-19, D-dimer, increased survival, Enoxaparin, Apixaban

## Abstract

All autopsy studies demonstrated widespread thrombosis and alveolar-capillary microthrombi as the cause of death among patients with COVID-19. The autopsy studies are the gold-standard for diagnostic accuracy and therapeutic strategies for any clinical scenarios. The author initially observed that patients already taking therapeutic dose of oral direct factor Xa inhibitors for an unrelated reason, have significantly better survival rates than those not taking any anticoagulants. This influenced the author to conduct a retrospective chart review of the hospitalized patients in Jackson Hospital (Alabama) to evaluate the effect of variable doses of anticoagulation among COVID-19 patients. The study found that serum inflammatory bio-marker D-dimer trends are associated with changes in oxygen requirement among patients with COVID-19, if patients present at an early stage, and titration of Enoxaparin (anticoagulation) dose based on D-dimer trends leads to increased patient survival.

## 1 Introduction

How does the widespread coagulation cascade gets initiated in the case of COVID-19? The COVID-19 infects cells through the upregulation of angiotensin-converting enzyme 2, which is considered to be the functional receptor for COVID-19 cellular entry [1]. This endothelial cell entry may cause microscopic cellular injury, possibly causing the endothelitis needed to initiate the cascade for anticoagulation. The basic concept of hemostasis (i.e., cessation of bleeding from the injured cellular site) involves cellular injury leading to platelet activation, generation of fibrin by activated coagulation factors, and inhibition of procoagulant factors to prevent excessive clot propagation. In addition, there is fibrinolysis to dissolve the fibrin clot. The microscopic endothelial injury is possibly the reason for the microscopic size of thromboembolism. An autopsy study showed a significantly increased severe endothelial injury (endotheliosis), widespread thrombosis with microangiopathy, and alveolar-capillary microthrombi in the lungs of the patients who died of COVID-19 compared to the lungs of individuals who died of influenza or other causes [2]. Another autopsy study of 21 individuals with COVID-19 showed prominent pulmonary emboli (PE) in 19% of cases and microthrombi in alveolar capillaries in 45% of cases. Interestingly, the primary cause of death in each of the cases was found to be respiratory failure due to exudative diffuse alveolar damage and massive capillary congestion accompanied by microthrombi [3]. Autopsy studies are considered the “gold standard” for diagnostic accuracy and diagnostic and therapeutic error prevention. Developing a treatment strategy to prevent mortality based on what is seen in the autopsy studies should be the guiding principle in developing treatments for COVID-19.

In addition, Ockham’s Razor, which implies that the simplest explanations accounting for all facts are more likely to be correct, can also shed light on finding the mechanism of COVID-19 infection under very diverse clinical presentations. Symptoms including fever, shortness of breath, and chest pain may be due to microthrombi in the heart and lungs capillary bed. Parallely, abdominal pain, diarrhea, and ischemic colitis may be due to microthrombi in the intestinal capillary bed. While the ARDS and PE may be due to the alveolar-capillary microthrombi and larger thrombi, stroke, seizures, and loss of sense of smell may be due to microthrombi in the capillary bed of the brain.

In the clinical setting, venous thromboembolism is seen in up to one-third of intensive care unit (ICU) patients with COVID-19, even when a prophylactic dose of anticoagulation is used. A recent study conducted on 44 ICU patients also found a high rate of thromboembolic events among the study subjects [4]. Patients with COVID-19 are reported as having numerous coagulation abnormalities, including elevated factor VIII, elevated fibrinogen, circulating prothrombotic microparticles, and hyper-viscosity [5,6]. Yet, the coagulation parameters in COVID-19 are distinct from Disseminated Intravascular Coagulation. In COVID-19, there is high fibrinogen and high factor VIII activity, indicating that the major consumption of coagulation factors is not occurring [5].

It is important to find a biological marker that can predict COVID-19 survival or death. This retrospective case series study focused on assessing the D-dimer trends as a candidate biomarker that is associated with the level of oxygen requirements and responsive to anticoagulation dose, among patients with COVID-19. The goal of this pilot study was to generate effective treatment strategies to prevent COVID-19 mortality and morbidity using traditionally available drugs and biomarkers.

## 2 Method

This study retrospectively evaluated the COVID-19 admission at Jackson Hospital, Alabama, USA from June 15th, 2020 to June 15th, 2021. The study considered the patients who received traditional treatments with Remdesivir, convalescent plasma, Plaquenil, intravenous steroids, and prophylaxis dose of Enoxaparin as a control group. Those patients who received a variable dose of anticoagulation (Apixaban, Enoxaparin, and Heparin) plus the IV Dexamethasone for treating COVID-19 were considered the treatment group.

The author’s previous publication did not assess anticoagulants’ variable effects in treating COVID-19 [7]. The goal of the retrospective pilot study was to evaluate the variable effects of different anticoagulants (like Eliquis and different doses of Enoxaparin [40 mg SubQ BID; 0.5 mg/kg SubQ BID; and 1 mg/kg SubQ BID]) to assess the survival benefit of COVID-19 while treated with anticoagulation. The retrospective study evaluated different modalities of anticoagulation treatments in the same patients sequentially to eliminate bias related to age, sex, race, immunological status, and genetic variations. The study evaluated step-up dosing for Enoxaparin therapy where Enoxaparin doses were titrated based on D-dimer levels. Any patient admitted to the ICU from the COVID-19 medicine floor was considered a major event. The study results were shown in charts with trends of D-dimer and corresponding oxygen requirements along with dose of Enoxaparin to generate study hypothesis. Patients presented variable D-dimers due to associated chronic medical conditions, and corresponding oxygen requirements were also variable, the study did not conduct a commutative analysis.


**Ethical approval:** The research related to human use has been complied with all the relevant national regulations, institutional policies, and in accordance the tenets of the Helsinki Declaration and has been approved by the authors’ institutional review board of Jackson Hospital, Montgomery, Alabama.
**Informed consent:** All patients were admitted to the hospital as they meet hospitalization criteria and gave signed consent to treatments with any FDA approved drugs.

## 3 Results

**Figure 1:**
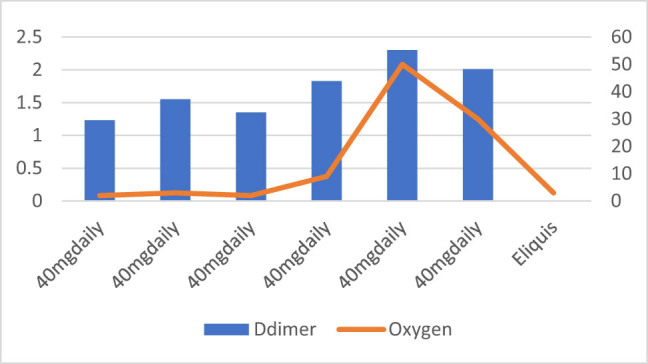
Patient number 1 presented at early stage requiring nasal canula oxygen and D-dimer level was also low on presentation. The figure demonstrates that increase or decrease in D-dimer trends is associated with increasing and decreasing trends of oxygen requirements. This patient was treated with low dose of Enoxaparin (40 mg SubQ daily) which is regular DVT prophylaxis dose.

**Figure 2:**
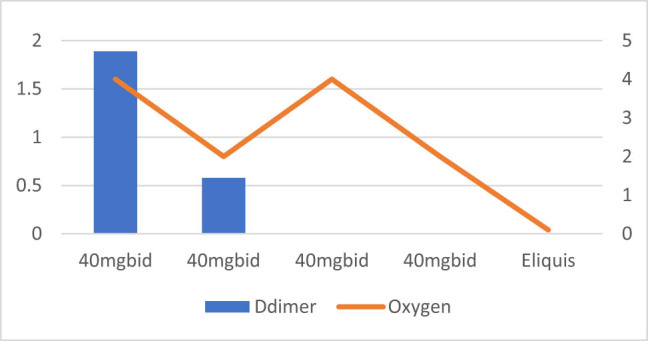
Patient number 2 presented at early stage requiring nasal canula oxygen and D-dimer level was also moderately low on presentation. The figure demonstrates that increase or decrease in D-dimer is associated with increasing and decreasing trends of oxygen requirements. In this case, only two D-dimer levels were available. This patient was treated with intermediate dose of Enoxaparin (40 mg SubQ BID) which is higher than DVT prophylaxis dose.

**Figure 3:**
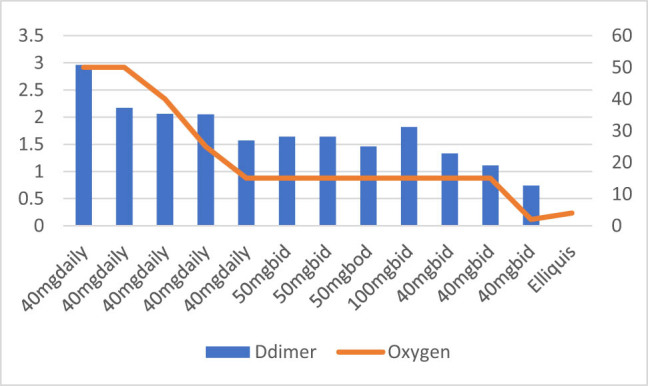
Patient number 3 presented at a later stage requiring high flow nasal canula oxygen and D-dimer level was also moderately high on presentation. The figure demonstrates that increase or decrease in D-dimer trends is associated with increasing and decreasing trends of oxygen requirements. This patient was treated with intermediate dose of Enoxaparin (40 mg SubQ BID) which was adjusted based on D-dimer trends. At some point, patient also received 100 mg SubQ BID dose of Enoxaparin due to increased D-dimer and patient responded appropriately and subsequently discharged home.

**Figure 4:**
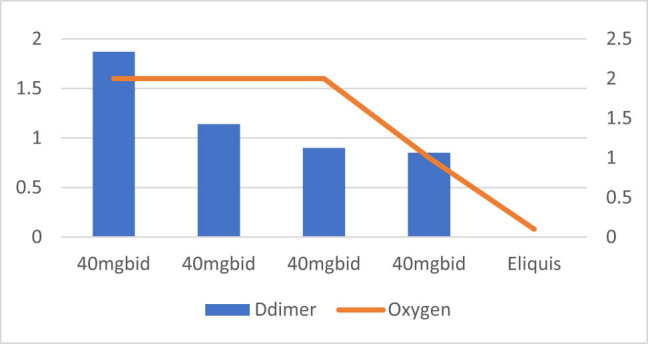
Patient number 4 presented at an early stage requiring nasal canula oxygen and D-dimer level was also moderately low on presentation. The figure demonstrates that increase or decrease in D-dimer trends is associated with increasing and decreasing trends of oxygen requirements. This patient was treated with intermediate dose of Enoxaparin (40 mg SubQ BID).

**Figure 5:**
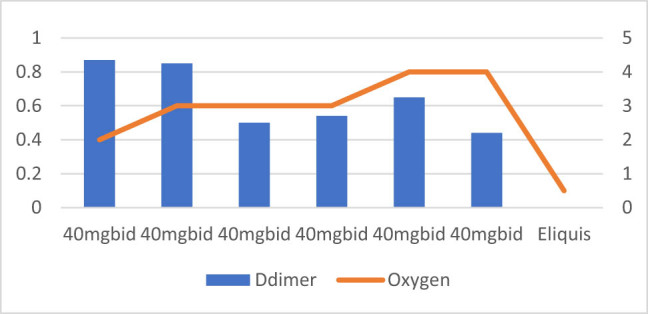
Patient number 5 presented at an early stage requiring nasal canula oxygen and D-dimer level was also low on presentation. The figure demonstrates that increase or decrease in D-dimer trends is associated with increasing and decreasing trends of oxygen requirements. This patient was treated with intermediate dose of Enoxaparin (40 mg SubQ BID).

**Figure 6:**
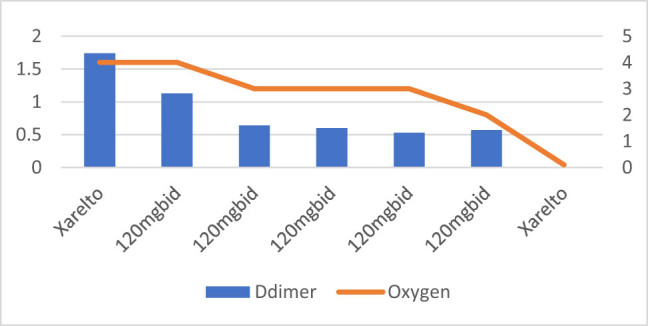
Patient number 6 presented at an early stage requiring nasal canula oxygen and D-dimer level was also low on presentation. The figure demonstrates that increase or decrease in D-dimer trends is associated with increasing and decreasing trends of oxygen requirements. This patient was on anticoagulation with Rivaroxaban (Xarelto). The patient was given the required oxygen, but was considered Rivaroxaban failure and converted to full dose of Enoxaparin. The patient was an obese patient, subsequently was discharged home with Rivaroxaban.

**Figure 7:**
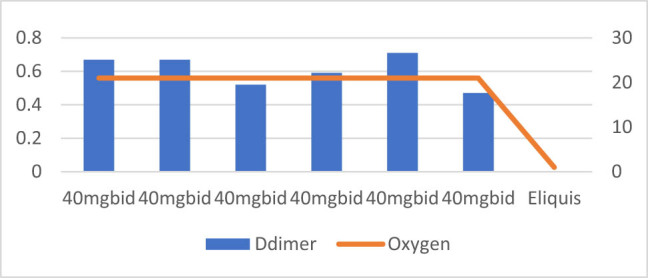
Patient number 7 presented at an early stage requiring oxy mask and D-dimer level was also low on presentation. The figure demonstrates that D-dimer trends slightly increase or decrease, but the increasing requirements remained almost same as patient was possibly getting high oxygen via oxy mask. This patient was treated with intermediate dose of Enoxaparin (40 mg SubQ BID).

**Figure 8:**
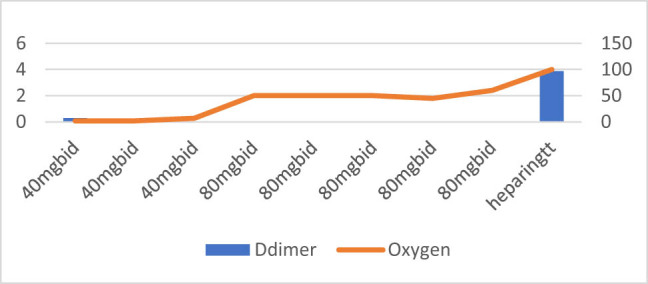
Patient number 8 presented at an early stage requiring nasal canula oxygen and D-dimer level was also low on presentation. The D-dimer levels are missing during the hospital stay, but the figure shows the increasing trends of oxygen requirements. The patient on admission had A Fib with rapid ventricular response which is also a hypercoagulable disease (like COVID-19) needing full anticoagulation from admission. There was delay of 72 h in increasing Enoxaparin doses (from 40 mg SubQ BID to 80 mg SubQ BID) leading to intra-aortic occlusion and subsequently patient expired.

**Figure 9:**
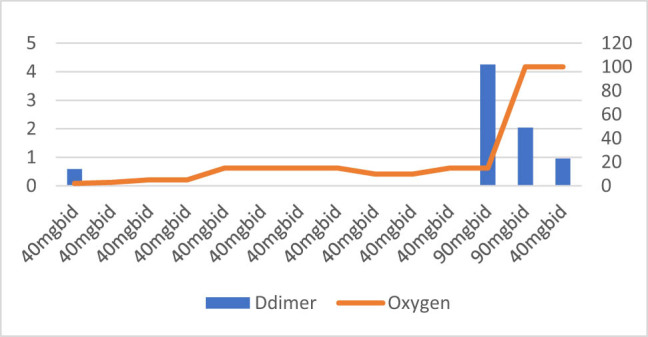
Patient number 9 presented at an early stage requiring nasal canula oxygen and D-dimer level was also low on presentation. The D-dimer levels are missing during the hospital stay except on admission and at the end. But the figure shows that the D-dimer increased significantly prior to intubation; however, it was possibly a missed opportunity as D-dimer was not checked in the interim. The patient was treated with intermediate dose of Enoxaparin (40 mg SubQ BID) since the admission and adjusted to full dose of Enoxaparin (90 mg SUBQ BID) when patient was intubated. This patent subsequently died due to COVID-19.

**Figure 10:**
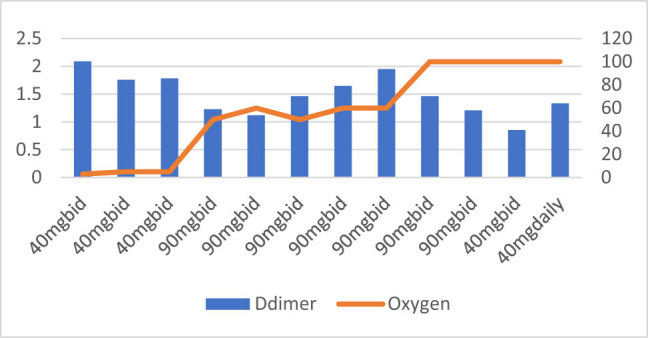
Patient number 10 presented at an early stage requiring nasal canula oxygen and D-dimer level was also moderately high (>2) on presentation. The figure demonstrates that initial D-dimer trends decreased, but the oxygen requirements remained low until day 3 and then started to increase following increasing trends of D-dimer. The Enoxaparin dose was adjusted on day 4, but patient developed pneumothorax and pneumopericardium as well as had ST elevation myocardial ischemia leading to patient’s death.

The study reviewed 100 COVID-19 patients treated at Jackson hospital, Alabama (United States) from June 15th, 2020 to June 15th, 2021. The retrospective case series analysis evaluated 100 patients, but chose to show only ten patients to demonstrate different modalities of treatment strategies. Figures 1–10 demonstrate the effect of anticoagulation, associated D-dimer, and corresponding oxygen requirements.

## 4 Discussion

Three large randomized clinical trials named ATTACC, ACTIV-4a, and REMAP-CAP were terminated early as the investigators found that among noncritically ill patients with Covid-19, an initial strategy of therapeutic dose of anticoagulation with heparin increased the probability of survival to hospital discharge with reduced use of cardiovascular or respiratory organ support as compared with usual-care thromboprophylaxis (reported on August 4, 2021) [8]. These three clinical trials also reported that among critically ill patients with COVID-19, an initial strategy of therapeutic dose of anticoagulation with unfractionated or low molecular weight heparin was not associated with a greater probability of survival to hospital discharge or a greater number of days free of cardiovascular or respiratory organ support than was usual-care pharmacologic thromboprophylaxis [9]. In previous publication also the author observed the same findings of patients with COVID-19 who were already on anticoagulation, even before the diagnosis of COVID-19, and published that those patients had almost near zero mortality rates due to COVID-19 [7]. These three clinical trial findings validate the study’s observation of why patients already on anticoagulation with Apixaban (or Rivaroxaban) were surviving as noncritically ill or even asymptomatic when they were taking anticoagulation orally.

Patients who were admitted at the hospital medical floor (noncritically ill) have significant mortality benefit with concomitant use of anticoagulation and intravenous steroids. However, those patients who were treated with anticoagulation in the early stage of COVID-19 and later moved from the hospital floor to the ICU have a higher chance of recovery compared to those who did not receive any anticoagulation prior to their critical care unit admission. The reason behind these failures is that anticoagulation does not dissolve microthrombi (blood clots), rather prevents their further growth. Treating patients with anticoagulation only can benefit when capillaries are still open (not clogged due to microthrombi). Bottom line is that patients presenting to hospital for need of nasal cannula oxygen or high flow nasal cannula oxygen and/or patient with low D-dimer, usually survive with anticoagulation and steroid combination treatments.

These COVID-19 hospital floor patients provide unique experiences because of their early stages of the disease presentation compared to patients admitted in the ICUs. The study observed that COVID-19 patients presenting in the later stages of COVID-19 were directly admitted to the ICU due to intubation or BiPAP requirement and were more likely to have poor outcomes compared to patients presenting at an earlier stage in the COVID-19 hospital floor.

According to this study, anticoagulation along with intravenous dexamethasone has significant mortality and morbidity benefits when patients are in the early stage of the disease process. The study observed that increasing trends of D-dimer in any patient were inversely proportional to the survival and/or clinical recovery from COVID-19 and *vice versa*. There is a dose-effect relationship between the decreasing trends of D-dimer and the used anticoagulation dose. That means, the quicker recovery is directly proportional to the higher amount of anticoagulation used for treating early-stage COVID-19. Hence, D-dimer can be considered to predict patient outcomes based on which healthcare facility needs to tackle COVID-19. The intravenous steroid (Dexamethasone) provided significant benefit when used concomitantly along with anticoagulation for the noncritically ill hospitalized patients. The steroid possibly helped reduce virus-induced immunological response. The study also observed that administering antiviral drugs (e.g., Remdesivir) or any drugs that suppress viral load (e.g., convalescent plasma) resulted in a negligible mortality benefit. It is possible that patients presented with respiratory failure, reducing the viral load swimming in the bloodstream, may not have an impact because there are millions of copies residing inside endothelial cells. Thus, the strategy to treat COVID-19 patients, when patients present at a later stage of the disease process, is to treat disease outcome (microthrombi) using therapeutic anticoagulation rather than trying to suppress the viral load (using Remdesivir or convalescent plasma or hydroxychloroquine). The study also observed patients who developed life-threatening bleeding while on anticoagulation related to COVID-19. Thus, one should thoroughly evaluate patients for risk of bleeding before starting therapeutic anticoagulation, discuss with the patient and family members about the risk of bleeding, and, if possible, temporarily hold aspirin and plavix, while treating the patient with therapeutic anticoagulation for COVID-19.

This study also observed a significant decrease in death rates attributed to ARDS, PE, stroke, or any other significant complications among all patients diagnosed with COVID-19 who are already on high dose of anticoagulation. These observations indicate that therapeutic anticoagulation may protect patients from microthrombi related COVID-19 complications. This is because anticoagulants work by interrupting the process involved in the formation of microthrombi and do not dissolve the microthrombi once they are formed. This mechanism of action also indicates that starting therapeutic anticoagulation after the diagnosis of COVID-19 may not work if widespread microthrombi are already formed prior to the initiation of anticoagulation. The study also observed that direct factor Xa inhibitors are superior to vitamin K antagonists (Warfarin) in terms of ARDS and other morbidity prevention (acute kidney injury). This difference may be related to the fact that direct factor Xa inhibitors prevent factor Xa from cleaving prothrombin to fibrinogen only, which might have a benefit over the blocking function of the vitamin K antagonists leading to depletion of all the vitamin K-dependent coagulation factors (factors II known as prothrombin, VII, IX, and X and protein C). It is unknown why Heparin, which works by enhancing the activity of antithrombin, and oral direct Xa inhibitors are less effective when compared to low molecular weight Heparin (Enoxaparin) among hospitalized patients with high D-dimer. It should be noted that Enoxaparin has 100 units of anti-factor Xa activity per mg, which might play a role in the case of COVID-19. The study also observed that if patients present with ARDS while on direct acting Xa inhibitors, switching to therapeutic dose of enoxaparin plus IV steroids provide a better chance of recovery from COVID-19.

The study observed that when patients present with a very high D-dimer, even therapeutic anticoagulation is less likely to prevent a catastrophic outcome. If a patient presents at a later stage like needing intubation or requiring BiPAP on admission, despite using therapeutic anticoagulation, they demonstrate very poor outcomes. In addition, this study found that patient on BiPAP for a prolonged period develop pneumothorax. Given that these critically ill COVID-19 patients do not respond to therapeutic anticoagulation, any clinical trial devoted to critically ill COVID-19 patients may fail to show benefits due to an overwhelming burden of microthrombi where anticoagulation does not dissolve it, rather help prevent getting those thrombi larger. To evaluate the effectiveness of anticoagulation, a clinical trial should compare the early stages of COVID-19 patients who are being treated on the hospital’s COVID-19 floor. This study observed a significant reduction in time to ventilation or reduction in mortality, if patients are given anticoagulation at an early stage of the disease process when the patients do not need critical care unit admission. Similar findings were also reported in the New England Journal of Medicine on August 4, 2021 [8].

However, the study observed that patients presenting with a very low D-dimer and a low basic metabolic index (BMI), would respond to a prophylactic dose of Enoxaparin (or intermediate dose) and show a significant reduction in mortality when compared with obese patients. This may be attributed to the therapeutic dose of Enoxaparin possibly being very close to the prophylactic dose, because of the patient’s lower weight. A similar pattern was also observed among patients on direct factor Xa inhibitors. The patient’s weight may be a significant factor in the prevention of ARDS. Drug doses need to be adjusted for the reduction in mortality, while patients are treated with therapeutic anticoagulation. However, low molecular weight heparin resulted in a better survival benefit compared to any oral anticoagulants across the spectrum of COVID-19 patients.

Any randomized controlled clinical trial might not show the benefit of anticoagulation, if the study subjects are critically ill and are not matched or randomized based on the patient’s BMI and the D-dimer level at the time of any enrollment. The study observed that the D-dimer level directly correlates with the critical versus noncritical stages of COVID-19 at the time of enrollment. Both factors (patient BMI and D-dimer) should be randomized among the treatment and control arms for a sound clinical trial to evaluate whether anticoagulation will show a survival benefit or not.

A quick study with a similar impact can be done by evaluating the patient population who are already taking a therapeutic dose of Apixaban, Rivaroxaban, or Betrixaban for chronic medical conditions like atrial fibrillation, DVT, PE, or mechanical valve. We need to evaluate whether this subset of patients develops any ARDS, stroke, PE, DVT, or has a decreased death rate when diagnosed with COVID-19. A previous study on laboratory-confirmed COVID-19 analyzed across the New York City health system did not show any survival (all-cause mortality) benefit and time to mechanical ventilation effect on patients who were already taking therapeutic anticoagulation unrelated to their COVID-19 diagnosis [10]. This study did not clarify what type of anticoagulants (direct Xa inhibitors, direct antithrombin, or vitamin K antagonists) were taken by patients and were not adjusted for patient weight to determine the oral anticoagulation failure. This study also observed frequent failure of Warfarin and direct factor Xa inhibitor among very obese patients. Therefore, it is likely that the New York study failed to show survival benefit due to failure to account for the patient weight (or obesity) and Warfarin related failures, as patients were almost always presented with subtherapeutic INR and morbidly obese patients failed due to inadequate dosing of oral anticoagulants.

The study observed the following treatment strategies have the highest survival benefits among patients with COVID-19:

(1) If any patient get diagnosed with COVID-19 but does not meet the hospitalization criteria, then treat the patient with direct Xa inhibitors (preferably, Apixaban 5 mg PO BID) for at least one month after considering bleeding risk related to therapeutic anticoagulation.
(2) If the patient is diagnosed with hypoxia on presentation, admit the patient to a hospital, and titrate Enoxaparin dose based on serum D-dimer trends. Consider low dose of Enoxaparin (40 mg SubQ daily) or intermediate dose of anticoagulation (Enoxaparin 0.5 mg/kg SubQ twice a day), if patients with COVID-19 have high bleeding risk.
(3) Consider starting Dexamethasone of 6 mg intravenous (IV) daily, concomitantly, if the patient requires oxygen.
(4) Consider starting full dose of anticoagulation among patients with COVID-19 who present with concomitantly another hypercoagulable disease.
(5) For the hospitalized patients, consider intermediate-dose of Enoxaparin (40 mg SubQ twice a day) among patients with high bleeding risk and titrate dose use based on D-dimer trends.
(6) Adjust enoxaparin doses based on kidney function. Consider Heparin drip for for any patient with end-stage renal disease instead of Enoxaparin.
(7) Increasing or decreasing trend of D-dimer accurately predicts changes in oxygen requirements within 24 h. D-dimer trends provide an early opportunity to titrate up or down Enoxaparin dose before the patient’s status changes. Patients with COVID-19 are less likely to die, if their D-dimer level is low.
(8) Calculate bleeding risk before starting therapeutic anticoagulation. Request permissions from patients and family members, when they are started on high-dose of anticoagulation as there is a bleeding risk.
(9) If patients on anticoagulation at home for an unrelated reason gets admitted to a hospital with COVID-19, start full dose of anticoagulation (Enoxaparin or heparin) instead of continuing home anticoagulation. In addition, start Dexamethasone of 6 mg (or 10 mg) IV daily, concomitantly.
(10) When patients with COVID-19 are ready for discharge, send the patient home with direct Xa inhibitors (preferably with Apixaban, not Coumadin) for at least one month to prevent DVT, PE, stroke, or post-COVID-19 syndrome.

## 5 Conclusion

Serum inflammatory bio-marker D-dimer trends are associated with changes in oxygen requirement among patients with COVID-19, if patients present at an early stage and titration of Enoxaparin (anticoagulation) dose based on D-dimer trends leads to increased patient survival. Recently published clinical trial validates the fundamental principles of the study’s ten steps COVID-19 treatment strategies. Starting with high dose anticoagulation upon diagnosis of COVID-19 among noncritically ill COVID-19 patients showed improved survival benefit. This study recommends starting direct factor Xa inhibitors, among patients who do not meet hospitalization criteria, to reduce the need for hospitalization and improve survival rates. The study’s rest of the steps for COVID-19 treatment strategies need to be validated through rigorous, well-designed clinical trials to reduce mortality and morbidity related to COVID-19.
